# Paper wasp nest-mediated biosynthesis of silver nanoparticles for antimicrobial, catalytic, anticoagulant, and thrombolytic applications

**DOI:** 10.1007/s13205-016-0459-x

**Published:** 2016-06-22

**Authors:** Agbaje Lateef, Monsurat A. Akande, Sunday A. Ojo, Bolaji I. Folarin, Evariste B. Gueguim-Kana, Lorika S. Beukes

**Affiliations:** 1 Laboratory of Industrial Microbiology and Nanobiotechnology, Department of Pure and Applied Biology, Ladoke Akintola University of Technology, PMB 4000, Ogbomoso, Nigeria; 2 Nanotechnology Research Group (NANO+), Ladoke Akintola University of Technology, PMB 4000, Ogbomoso, Nigeria; 3 Department of Microbiology, School of Life Sciences, University of KwaZulu-Natal, Private Bag X01, Scottsville, PieterMaritzburg, 3209 South Africa; 4 Microscopy and Microanalysis Unit, School of Life Sciences, University of KwaZulu-Natal, Private Bag X01, Scottsville, PieterMaritzburg, 3209 South Africa

**Keywords:** Wasp, Biosynthesis, Silver nanoparticles, Antimicrobial activity, Malachite green, Thrombolysis, TEM

## Abstract

Biosynthesis of silver nanoparticles (AgNPs) using nest extract of paper wasp (*Polistes* sp) was investigated in this work. The AgNPs were characterized by UV–Vis spectroscopy, Fourier transform infrared spectroscopy (FTIR), and transmission electron microscopy (TEM), and evaluated for antibacterial, antifungal, dye degradation, blood anticoagulation, and blood clot dissolution (thrombolytic) activities. The crystalline polydispersed AgNPs with size range of 12.5–95.55 nm absorbed maximally at 428 nm and showed anisotropic structures of sphere, triangle, hexagon, rod, and rhombus. The FTIR data showed prominent peaks at 3426 and 1641 cm^−1^, which indicate the involvement of phenolics compounds and proteins in the synthesis of AgNPs. The prominence of Ag in the EDX spectra showed that indeed, AgNPs were formed. The AgNPs showed potent antibacterial activities (12–35 mm) against three multi-drug strains of *Pseudomonas aeruginosa* and *Klebsiella granulomatis.* While the growth of *Aspergillus flavus* and *Aspergillus niger* was completely suppressed, the AgNPs produced growth inhibition of 75.61 % against *Aspergillus fumigatus* at 100 µg/ml. Furthermore, the AgNPs degraded malachite green to the tune of 93.1 %. The AgNPs also prevented coagulation of blood, while it completely dissolved preformed blood clots within 5 min showing the potent anticoagulation and thrombolytic activities. This study, which is the first of its kind to use nest extract of paper wasp for the synthesis of nanoparticles, has shown that the biosynthesized AgNPs could be deployed for biomedical and catalytic applications.

## Introduction

The art of synthesis and the applications of nanoparticles have continued to expand due to the abundance of biological materials that are rich in bioreductant molecules for the eco-friendly synthesis of nanoparticles. The avoidance of use of hazardous procedures has also widen the scope of biomedical applications, with appreciable level of biocompatibility and lesser toxicity compared with those synthesized using chemical route. To this extent, several biomaterials from plants, bacteria, fungi, algae, and among others have been used to produce nanoparticles (Kumar et al. 2012; Roopan et al. 2013; Velayutham et al. 2013; Das et al. 2014; Kumar et al. 2014; Metuku et al. 2014; Lateef et al. 2015a, b, c, 2016a, b; Lateef and Adeeyo 2015; Waghmare et al. 2015; Yugandhar et al. 2015). To expand the frontiers of applications of biomolecules for the green synthesis of nanoparticles, some recent studies have focused on the use of metabolites from arthropods (Xu et al. 2013; Aramwit et al. 2014; Lateef et al. 2015d). Among several metallic nanoparticles, silver nanoparticles (AgNPs) have been the most studied due to the versatility of its applications for diverse biological, biomedical, catalytic, electrical, and electrochemical purposes. These have made AgNPs to be of high relevance in environmental, catalytic, healthcare, food, agriculture, biomedical, and textile applications (Keat et al. 2015).

Paper wasps of the genus *Polistes* have been the most studied among the eusocial wasps (Hymenoptera, Vespidae, Polistinae), of which about 200 species of cosmopolitan occurrence had been described (West-Eberhard 2006; Sinzato et al. 2011; Nguyen and Kojima 2014). *Polistes* make colonies usually consisting of about 100 individuals that live in non-enveloped nests, which are made on twigs, leaves, dense shrubs and grass, hollow trees and elevated natural cavities, and manmade structures (Wenzel 1996; Clapperton and Lo 2000). The nests, which are paper-like in form, are derived from the foraging activities of wasps on plant materials (Evans and West-Eberhard 1970), and served as compartments to oviposit eggs by young females. In addition to richness in cellulose, the nests of *Polistes* have reportedly being rich in proteins with more than 20 amino acids detected from field and laboratory nests, of which glycine, serine, alanine, valine, and proline were the most prevalent amino acids (Singer et al. 1992; Kudô et al. 2000a, b). Though arthropods are abundantly rich in the synthesis of novel biomaterials, such as silk, cobwebs, and nests, the use of metabolites from arthropods for the green synthesis of nanoparticles seems to be at infancy with a few literature reports (Xu et al. 2013; Aramwit et al. 2014; Lateef et al. 2015d). Therefore, there is need to explore arthropods for their nanobiotechnological applications.

In our previous study, we have shown that a similar biomaterial, spider cobweb could be used for biosynthesis of AgNPs with potent antimicrobial activities (Lateef et al. 2015d). Therefore, this work was conceptualized to evaluate the potential of nest of paper wasp for the green and eco-friendly synthesis of AgNPs, thereby finding a biotechnological utilization of the biomaterial. The study further examined the antibacterial, antifungal, malachite degradation, blood anticoagulation, and thrombolytic (lysis of blood clots) activities of the biosynthesized AgNPs. To the best of our knowledge, this is the first report of the use of paper wasp’s nest for the biosynthesis of AgNPs.

## Materials and methods

### Preparation of paper wasp nest extract

Nests of paper wasp were obtained from a residential building in Ogbomoso, Southwest Nigeria. The nests were taken to the laboratory and washed thoroughly using distilled water to remove dust and other extraneous materials. The washed nest was allowed to air-dry at room temperature (30 ± 2 °C) and kept in air-tight container until further use. The modified method of Tszydel et al. (2009) as previously reported (Lateef et al. 2015d) was used to hydrolyze the nests. About 0.1 g of the nest was weighed and hydrolyzed with 10 ml of 0.1-M NaOH at 90 °C for 1 h. The hydrolyzed nest was allowed to cool, centrifuged at 4000 rpm for 30 min, and used without further purification.

### Biosynthesis and characterization of AgNPs

Biosynthesis of AgNPs was carried out as earlier reported (Lateef et al. 2015d) by reacting 1 ml of nest extract with 40 ml of 1-mM silver nitrate (AgNO_3_) solution for the reduction of silver ion. The reaction was carried out in static condition at room temperature (30 ± 2 °C) for 30 min. AgNPs biosynthesis was monitored through visual observation of the change of color and measurement of the absorbance spectrum of the reaction mixture using UV–Vis spectrophotometer (Cecil, USA).

Fourier transform infrared (FTIR) spectroscopy analysis was carried out using IRAffinity-1S spectrometer (Shimadzu, UK) on the powder sample of AgNPs. The AgNPs solution was centrifuged at 10,000 rpm for 20 min. The solid residue obtained was then dried at room temperature, and the powder obtained was used for FTIR measurement using KBr pellets.

The transmission electron microscopy (TEM) micrograph was obtained as follows. A drop of nanoparticles in suspension was placed on a 200-mesh hexagonal copper grid (3.05 mm) (Agar Scientific, Essex, UK) coated with 0.3 % formvar dissolved in chloroform. The particles were allowed to settle for 3–5 min on the grid, the excess liquid flicked off with a wick of filter paper, and the grids were then air dried before TEM viewing. Micrograph was obtained using a JEM-1400 (JEOL, USA) operating at 200 kV.

### Antimicrobial activities of synthesized AgNPs

The antimicrobial investigations involved the use of *Klebsiella granulomatis* and *Pseudomonas aeruginosa,* which were obtained from LAUTECH Teaching Hospital, Ogbomoso, and fungal strains of *Aspergillus niger, Aspergillus flavus,* and *Aspergillus fumigatus*. For antibacterial evaluation, each bacterium was grown overnight in peptone water, and 18 h-culture (~1 × 10^6^ cfu/ml) was used to seed the plates of Mueller–Hinton Agar (Lab M Ltd.). Thereafter, the plates were then bored using cork borer (7 mm) to create wells, to which 100 µl of graded concentrations (10–100 µg/ml) of AgNPs prepared by dispersion in sterile distilled water were added. The plates were incubated at 37 °C for 24 h, for the examination of zones of inhibition, which were measured. The antifungal activities of the biosynthesized AgNPs were evaluated using the methods of Khatami et al. (2015) by incorporating graded concentrations of AgNPs into potato dextrose agar plates, which were then inoculated with agar plug of 6 mm of 48-h old cultures of *Aspergillus niger*, *Aspergillus flavus,* and *Aspergillus fumigatus.* In the control experiments, fungal plugs were inoculated on PDA plates without the incorporation of AgNPs. All the plates were incubated at 28 ± 2 °C for 72 h. The diameters of fungal growths in all the plates were measured and used to determine the percentage growth inhibitions as follows: $\frac{{D_{\text{control}} - D_{\text{test}} }}{{D_{\text{control}} }} \times 100 \,\%$ where *D* is the diameter of fungal growth on the PDA plates.

### Dye-degradation activities of AgNPs

This was investigated through the decolorization of malachite green, by reacting 1 ml of the biosynthesized AgNPs at concentration of 20 and 40 μg/ml with 9 ml of 40 ppm of malachite green, while the control experiment consisted of 10 ml of the dye only. All the reaction vessels were subjected to agitation on a rotary shaker at 100 rpm for up to 24 h under ambient temperature of 30 ± 2 °C. The absorbance values of the reaction mixtures were determined using UV–Vis spectrophotometer at wavelength of 619 to calculate the percentage dye degradation as follows: ${\text{Percentage dye degradation }} = \frac{{A_{\text{control}} - A_{\text{test}} }}{{A_{\text{control}} }} \times { 1}00 \, \%$ where *A* is the absorbance at 619 nm.

### Anticoagulant and thrombolytic activities of biosynthesized AgNPs

The anticoagulant activity of the AgNPs was investigated by mixing 0.5 ml of 150 μg/ml of nanoparticles with 5 ml of freshly collected human blood from a healthy volunteer and then held at ambient condition (30 ± 2 °C) to observe visually for the coagulation of blood. In the control experiments, blood samples collected in EDTA bottle, and in ordinary clean bottle served as positive and negative control, respectively. The method of Harish et al. (2015) was used for the thrombolytic assay. Blood clot formed in vitro was spread on clean, grease-free glass slides, and then treated with 0.2 ml of AgNPs. This was monitored for the dissolution of the clot. The control experiments consisted of untreated blood clots, and that treated with AgNO_3_ solution and the nest-extract only. After visual observation, both anticoagulation and thrombolytic activities of the biosynthesized AgNPs were assessed through optical microscopy by capturing images of blood samples smeared on slides on an Olympus microscope.

## Results and discussion

### Biosynthesis and characterization of AgNPs

**Fig. 1:**
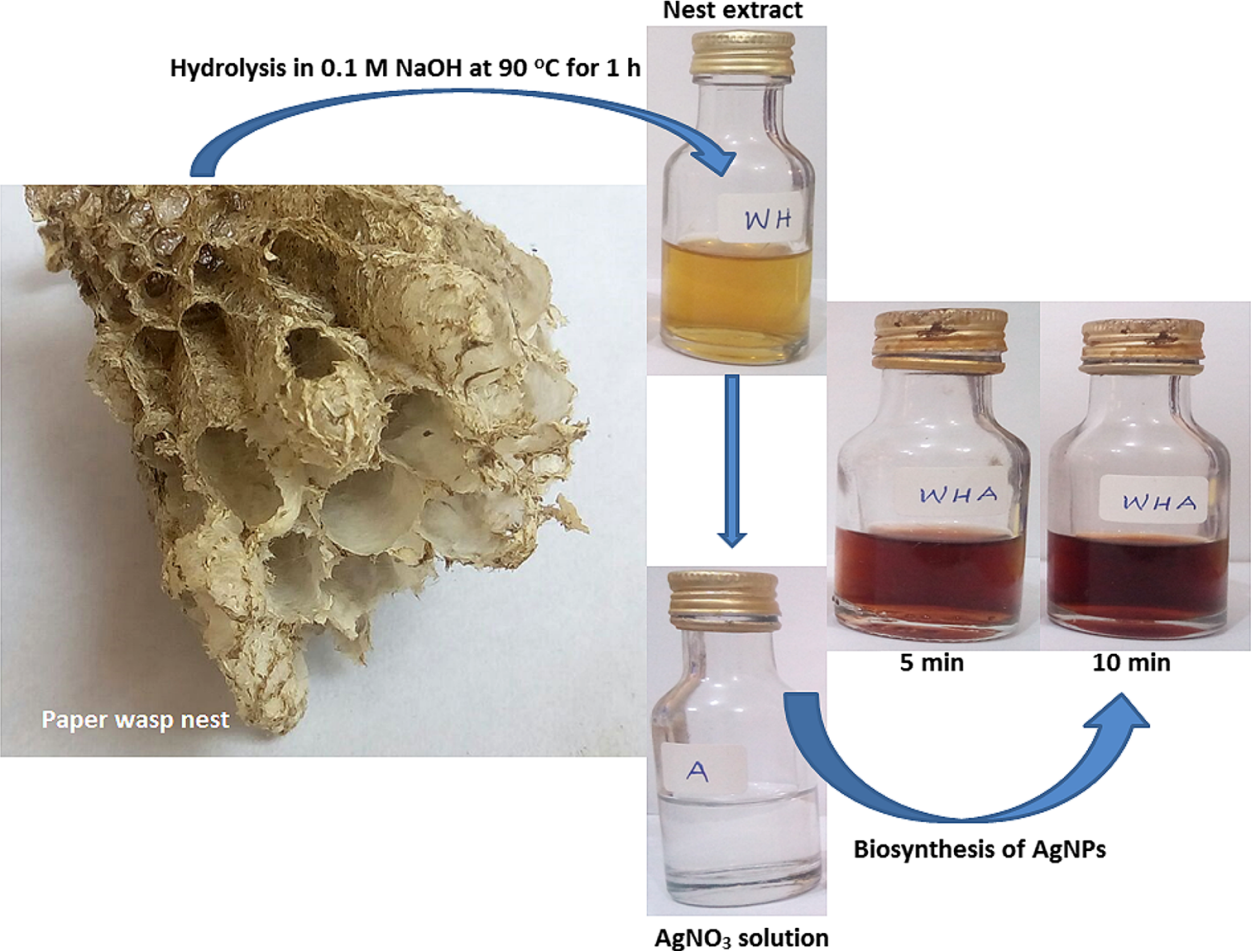
The biosynthesis of AgNPs using paper wasp nest extract

The nest extract with pH of 10.5 catalyzed the bioreduction of silver ion to produce metallic AgNPs with pH of 8.3 within 5 min. The AgNPs was brownish in color which stabilized after 10 min of reaction (Fig. 1). Several authors have reported color formation ranging from light yellow, yellow brown to dark brown for colloidal AgNPs (Das et al. 2014; Emeka et al. 2014; Lateef et al. 2015a, b, c, d; Netala et al. 2015) due to variations in the composition of bioreductant molecules which influence the surface plasmon resonance. Reports have indicated that nests of paper wasps are rich in proteins with abundant presence of amino acids (Singer et al. 1992; Kudô et al. 2000a, b) which could readily serve as bioreductant molecules for the green synthesis of AgNPs. In an earlier work, we established that proteinous molecules in the spider cobwebs were responsible for the green synthesis of AgNPs (Lateef et al. 2015d). Therefore, this work has further shown that metabolites of paper wasps can find application in the synthesis of nanoparticles.

**Fig. 2:**
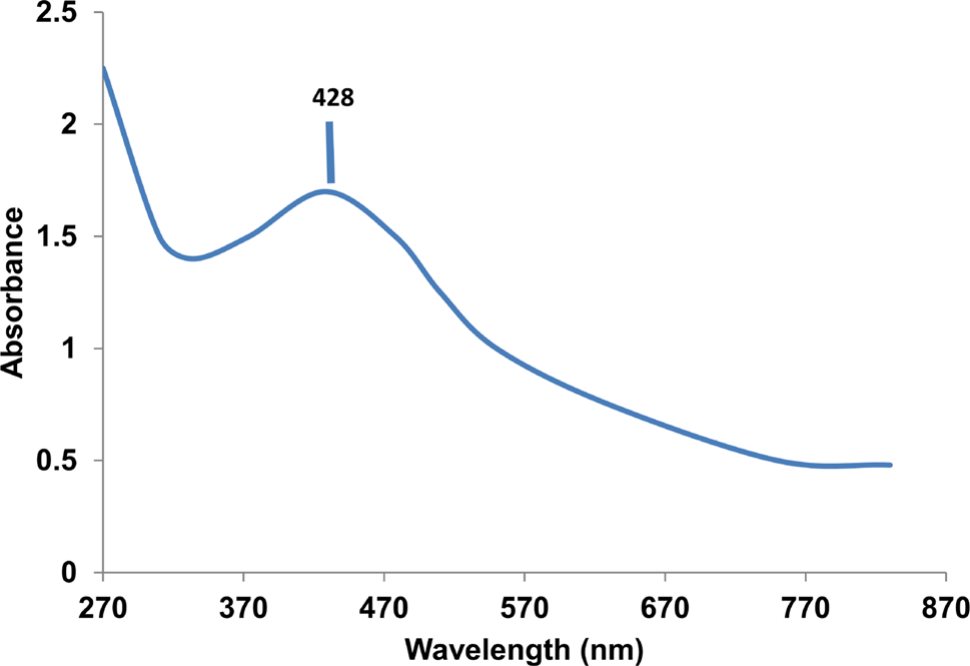
UV–Vis spectrum of the biosynthesized AgNPs

**Fig. 3:**
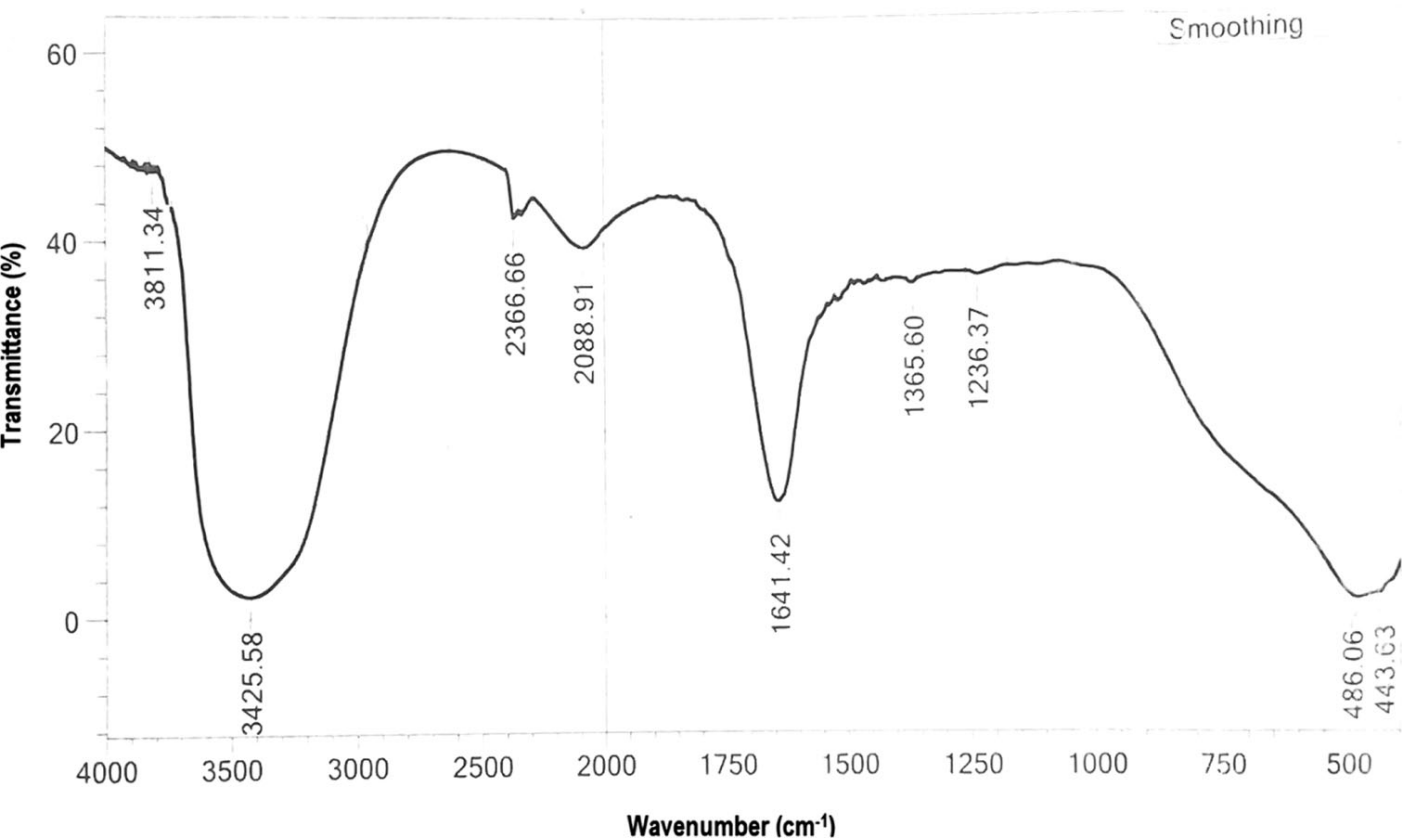
FTIR spectrum of the biosynthesized AgNPs

The colloidal AgNPs absorbed maximally at 428 nm (Fig. 2) with a broad peak that may depict polydispersed nanoparticles. The observed maximum absorbance falls within the range previously reported (Emeka et al. 2014; Anandalakshmi et al. 2015; Kathiraven et al. 2015; Lateef et al. 2015b, d). In the FTIR studies, prominent peaks were obtained at 3426 and 1641 cm^−1^, in addition to minor peaks at 3811, 2367, 2089, 1365, 1236, 486, and 444 cm^−1^ (Fig. 3). The major peaks at 3426 and 1641 cm^−1^ correspond to N–H of amines or O–H stretch of carboxylic acid, C=C stretch of alkenes or C=O stretch of amides, respectively (Emeka et al. 2014; Shankar et al. 2014) indicating the involvement of phenolics and proteins in the biosynthesis of AgNPs. While the presence of amino acids in the nests of paper wasps has been fully established (Singer et al. 1992; Kudô et al. 2000a, b), the occurrence of phenolics is unexpected, since the nests are largely produced from plant-based materials. It can, therefore, be concluded that proteins and phenolics present in the nest extract served as both capping and stabilization molecules in the synthesis of AgNPs.

**Fig. 4:**
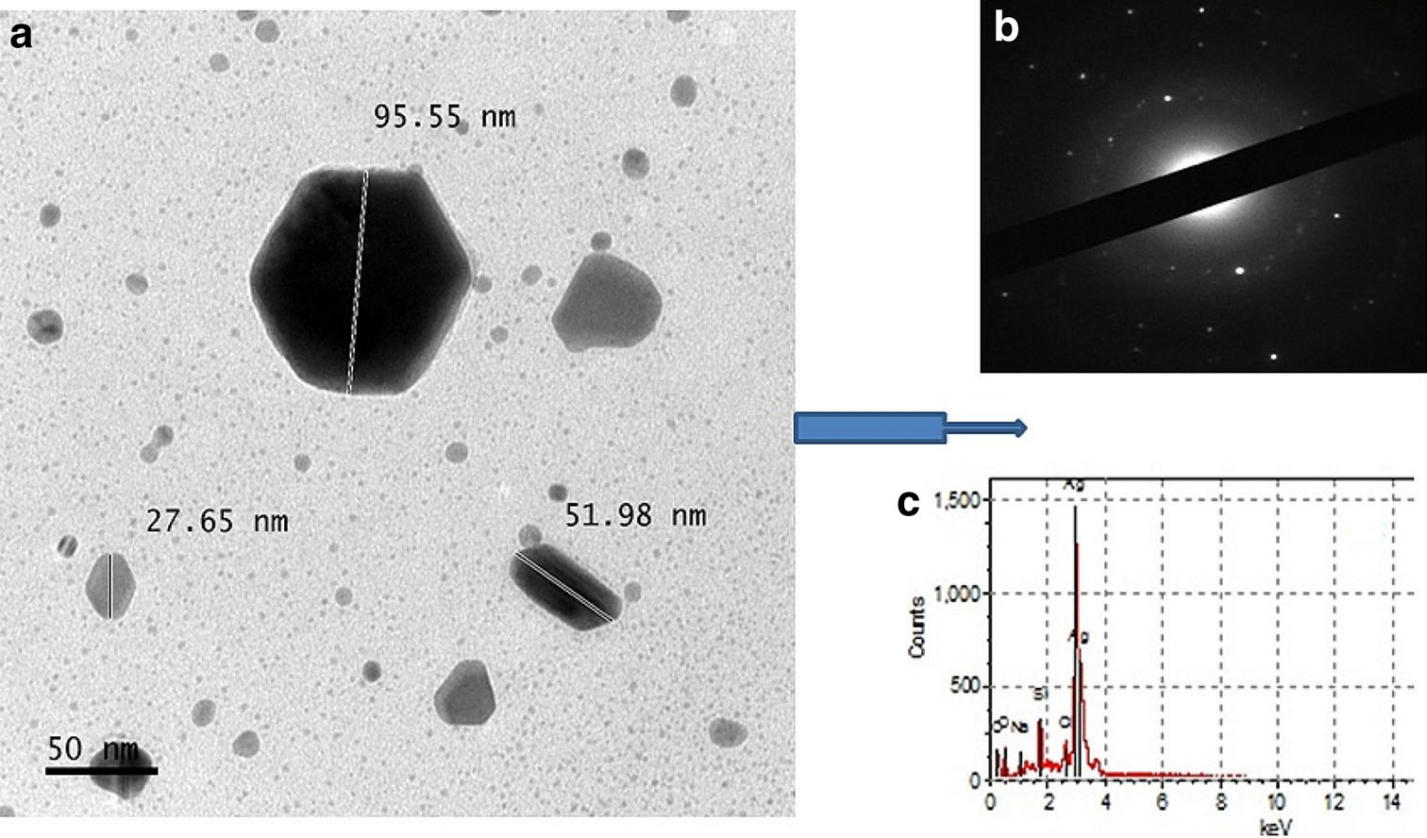
Transmission electron micrograph (**a**), SAED pattern (**b**), and EDX spectrum (**c**) of the biosynthesized AgNPs

The TEM result showed the formation of anisotropic structures with size range of 12.5–95.55 nm (Fig. 4a), which are well dispersed without any form of aggregation. The size range fully supports the polydispersed nature of the particles as shown in the broad band peak of the UV–Vis spectrum. Such AgNPs structures as hexagon, rhombus, 3-D triangle, rod and sphere were formed by the nest extract, which may be indicative of the richness and diversity of biomolecules present in the nest. Although green synthesis of spherical AgNPs abound in the literature, some authors have reported the formation of other shapes, such as triangle, pyramid, pentagon, hexagon, and other irregular ones (Vigneshwaran et al. 2006; Saifuddin et al. 2009; Rajakumar and Rahuman 2011; Santhoshkumar et al. 2011; Silambarasan and Jayanthi 2013). The selected electron area diffraction (SAED) showed that the AgNPs had crystalline nature (Fig. 4b) typical of Ag as shown by the ring-like pattern (Shankar et al. 2014), while the energy dispersive X-ray study showed the prominent presence of Ag (Fig. 4c).

### Antimicrobial activities of AgNPs

**Fig. 5:**
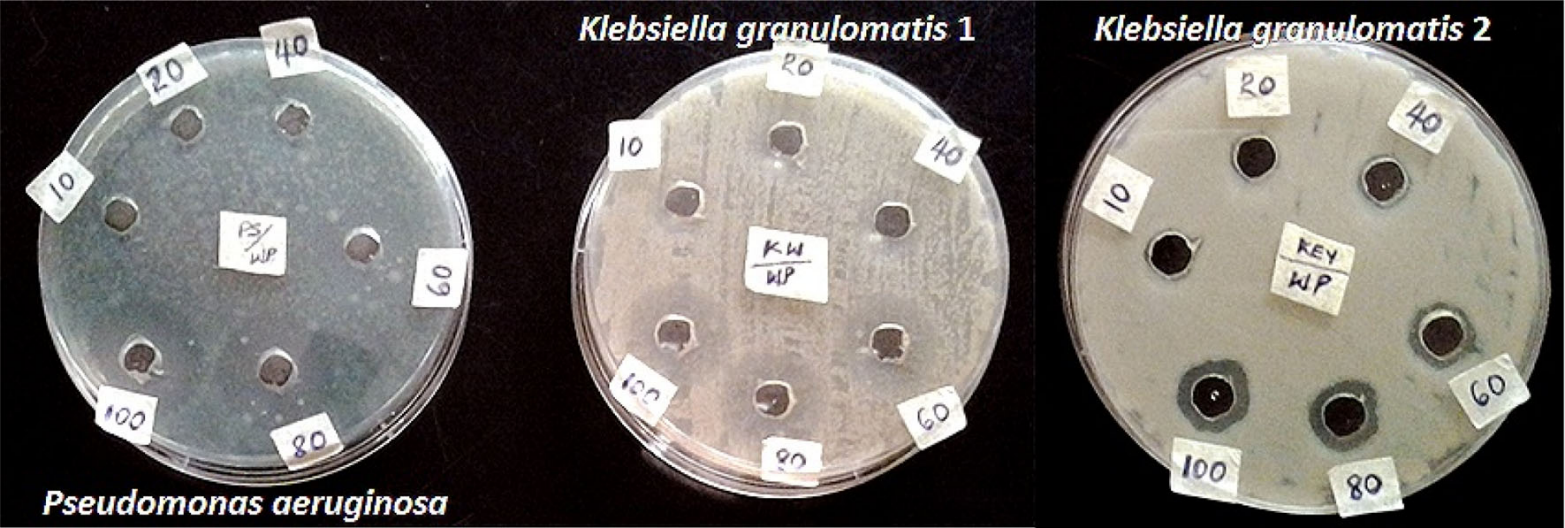
Antibacterial activities of biosynthesized AgNPs

The biosynthesized AgNPs showed potent antibacterial and antifungal activities on some selected strains. At concentrations of 60, 80, and 100 µg/ml, the AgNPs depicted antibacterial activities in the range of 12–35 mm against multi-drug resistant isolates of *Klebsiella granulomatis* and *Pseudomonas aeruginosa* (Fig. 5). The activities shown by the AgNPs are consistent with several reports on the antibacterial activities of AgNPs (Shankar et al. 2014; Emeka et al. 2014; Kathiraven et al. 2015; Anandalakshmi et al. 2015; Lateef et al. 2015a, b, c, d; Waghmare et al. 2015). The antibacterial activities of AgNPs have been linked to the interaction of AgNPs with sulfur and phosphorus containing constituents of the bacterial cell to initiate cell killing by attacking the respiratory chain and cell division (Mahendra et al. 2009). The antibacterial activity of the AgNPs against multi-drug resistant strains is noteworthy, in view of widespread occurrence of multi-drug resistant bacteria in diverse samples from the environment (Lateef 2004; Lateef et al. 2007, 2010; Lateef and Ojo 2016). Therefore, the potency of the AgNPs can enhance its application in combating drug-resistant bacteria.

**Fig. 6:**
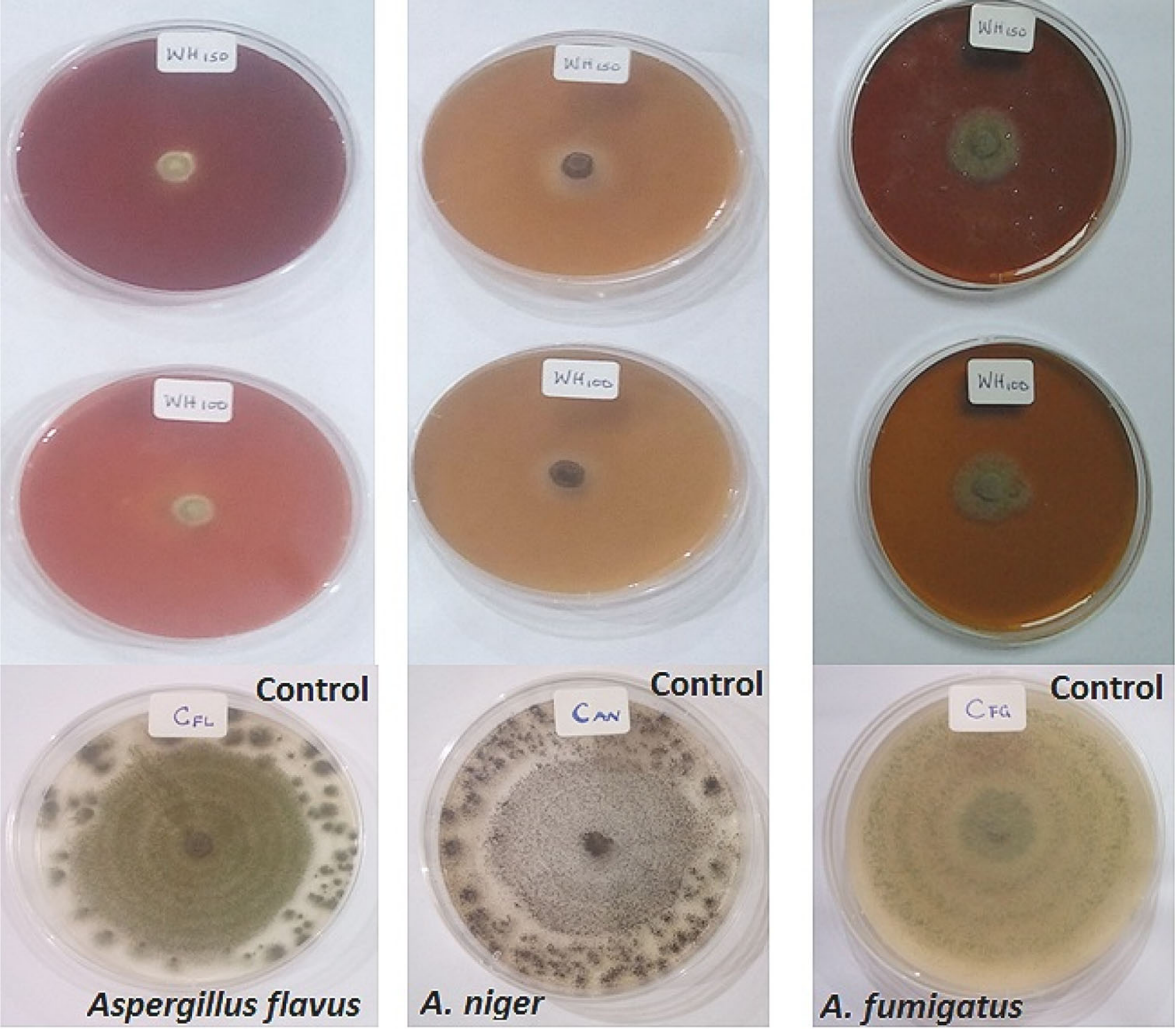
Antifungal activities of biosynthesized AgNPs

The AgNPs completely inhibited the growth of *Aspergillus niger* and *Aspergillus flavus* at tested concentrations of 100 and 150 µg/ml, whereas fungal growth inhibition of 75.61 % was achieved with *Aspergillus fumigatus* (Fig. 6). These observations are in contrast to the profuse growth that were obtained on the control plates. The antifungal activities agree with previously published results (Khatami et al. 2015; Ojo et al. 2016; Netala et al. 2015). The results obtained in this study have shown that the biosynthesized AgNPs can be used as potent antifungal agents.

### Degradation of malachite green using AgNPs

**Fig. 7:**
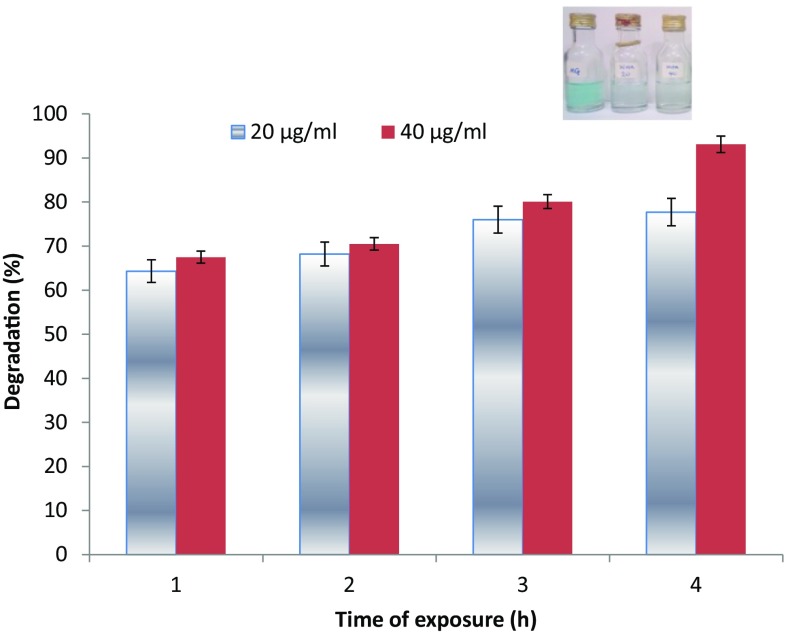
Degradation of malachite green by biosynthesized AgNPs

At concentrations of 20 and 40 µg/ml, the biosynthesized AgNPs showed degradation of malachite green in the range of 64.3–93.1 % during the period of 1–24 h (Fig. 7). The use of nanoparticles for the catalytic degradation of dyes has gained tremendous attention, as it is more advantageous than conventional methods, such as absorption, adsorption, coagulation, flocculation, ultrafiltration, reverse osmosis, and membrane technologies, that merely concentrate or transfer organic compounds from one phase to another (Soltani et al. 2012). The catalytic degradation of dyes by nanoparticles is achieved by serving as electron transfer mediators between active biomolecules borne on particles and dyes in a process termed electron relay effect (Gupta et al. 2011). The results obtained in this study have further showed that biosynthesized AgNPs could be deployed for the catalytic degradation of dyes in industrial wastewaters.

### Anticoagulant and thrombolytic activities of AgNPs

**Fig. 8:**
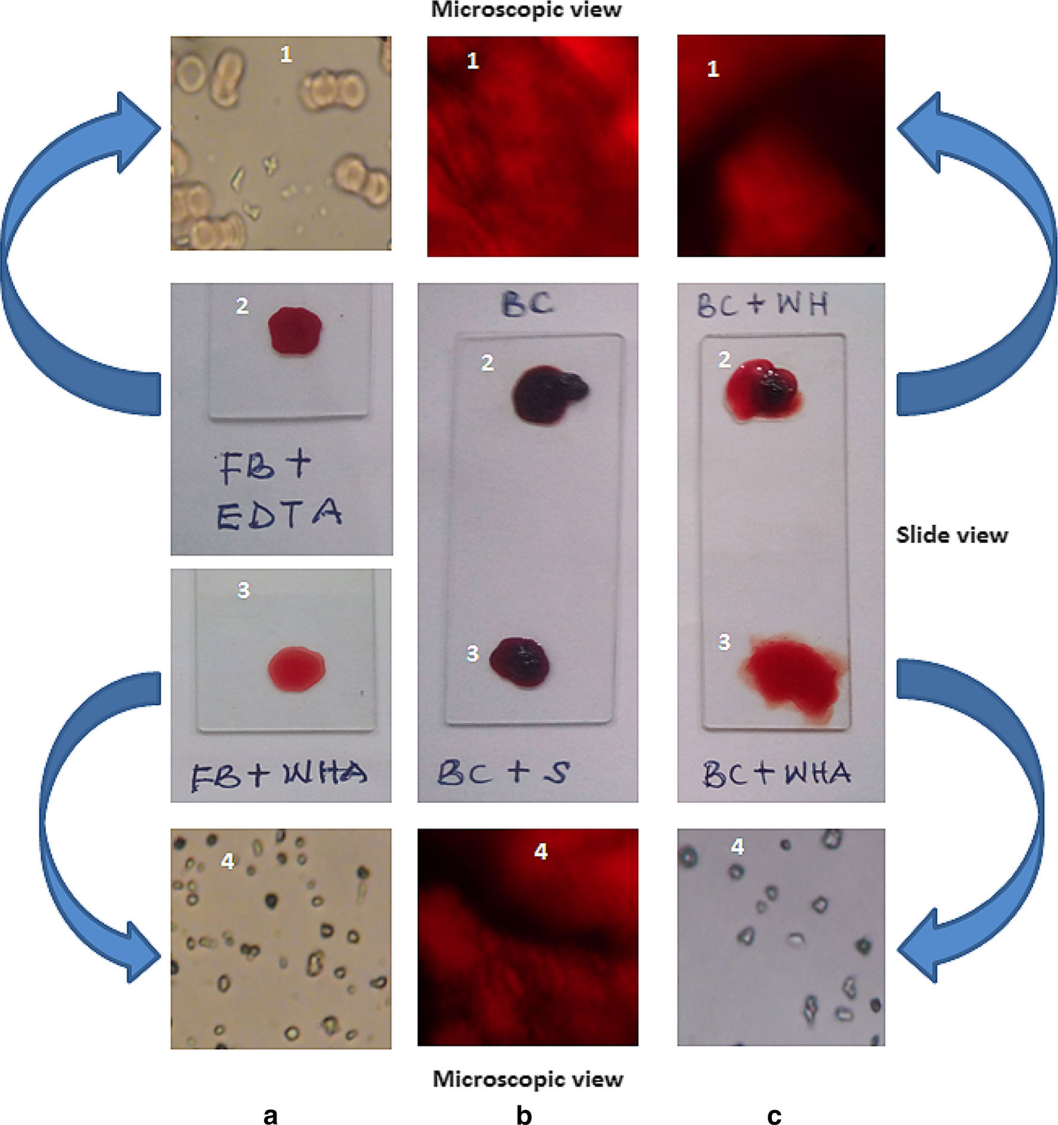
Anticoagulant (**a**), and thrombolytic activities (**b**, **c**) of biosynthesized AgNPs (*FB* fresh blood, *EDTA* anticoagulant, *WHA* wasp nest extract AgNPs, *BC* blood clot, *S* AgNO_3_ solution, *WH* wasp nest extract only)

The AgNPs showed potent blood anticoagulation and thrombolytic activities from the slide and microscopic observations (Fig. 8a–c). The AgNPs prevented formation of blood clot when used as anticoagulation agent (Fig. 8a3), which compared favorably with the positive control using EDTA (Fig. 8a2). Microscopic examination depicted dispersed red blood cells in EDTA-treated blood (Fig. 8a1) and AgNPs-treated blood (Fig. 8a4). The blood coagulation system is important to maintain steady blood flow, forestall bleeding and assists the innate immune system to prevent the spread of infectious agents (Esmon et al. 2011). This is not without a disadvantage, as the formation of blood clot arising from infection can damage tissues and leads to organ failure (Levi et al. 2010), often associated with cardiovascular disorders, autoimmune reactions, allergic responses, injuries, and emergence of cancer (Prandoni et al. 2007; Davalos and Akassoglou 2012). In addition, cancer cells can trigger blood coagulation through several processes, such as induction of proinflammatory cytokines, expression of procoagulant molecules on their surfaces and interaction with blood platelets (Jurasz et al. 2004; ten Cate and Falanga 2007).

In view of the complications that may arise from the blood coagulation disorders, it has become necessary to control the blood coagulation system to maintain a healthy state. This involves the prevention of aggregation of platelets, which can be achieved by AgNPs as previously demonstrated (Shrivastava et al. 2009) to inhibit the formation of thrombus. The non-toxicity of AgNPs to platelets and its profound antimicrobial activities can open a new regime of treatment of thrombosis. It has been shown that nanoparticles can impact varying degrees of influence on the blood coagulation system as a result of their size, charge, shape, and composition (Ilinskaya and Dobrovolskaia 2013). In addition to the sole use of AgNPs and perfluorocarbon emulsion (Lowe 2000) as antiplatelet agents, nanoparticles, such as gold (Hsu et al. 2011; Shiang et al. 2011) and iron oxides, can be used as carriers of active drugs to reduce the limitations of short half-life, prohibitive cost, and incidence of bleeding and hemorrhagic stroke in the prevention of coagulation of blood. The reformulation of traditional drugs such as heparin onto nanotechnology carrier platforms has potential to achieve reduced dosage, increased drug stability, increased shelf-life, and overall reduction in the cost of treatment (Ilinskaya and Dobrovolskaia 2013). It can, therefore, be inferred that the potency shown by AgNPs obtained in this study can have useful applications in nanomedicine for the prevention of blood coagulation.

The AgNPs dissolved the preformed blood clot on slides almost instantaneously with very high thrombolytic activity (Fig. 8c3), whereas the negative controls of AgNO_3_-treated (Fig. 8b3) and nest extract-treated (Fig. 8c2) blood clots did not lead to lysis of the blood clot. The result obtained in this study is similar to thrombolytic activity of xylan-mediated AgNPs as reported by Harish et al. (2015). Furthermore, microscopic views of the samples showed clear dispersion of the blood clot by AgNPs (Fig. 8c4), unlike the negative controls (Fig. 8b1, b4, c1). While blood clotting is necessary to curb excessive bleeding, its timely dissolution is equally important to prevent thrombosis and maintain homeostasis (Ilinskaya and Dobrovolskaia 2013). The timely and efficient dissolution of blood clot are key factors in achieving desirable outcome in patients with ischemia (inadequate supply of blood to organs) (Ilinskaya and Dobrovolskaia 2013), thereby necessitating optimization of the treatment regimes using nanotechnology. The traditional antithrombotic treatments, such as streptokinase, have such limitations in their suitability for application, including short half-life, neutralization of the foreign agents by antibodies, and danger of excessive bleeding. Interventions such as use of liposomes that can resist rapid degradation have been used as carriers of streptokinase with improved thrombolytic activity (Vaidya et al. 2011). Though there is limited information on the use of AgNPs as thrombolytic agent, the report herein presented is a further proof to the potential application of biogenic AgNPs as thrombolytic agent in the management thrombosis. We have also recently showed that Au and Ag–AuNPs possessed anticoagulant and thrombolytic activities (Ojo et al. 2016; Lateef et al. 2016c), with potential for biomedical applications.

## Conclusion

This study has, for the first time, demonstrated the eco-friendly synthesis of AgNPs using the nest extract of paper wasp. The biosynthesized AgNPs which absorbed maximally at 428 nm were polydispersed in nature with the size range of 12.5–95.55 nm, and showed anisotropic structures of sphere, triangle, hexagon, rhombus, and rod without any form aggregation. The crystalline AgNPs displayed remarkable antimicrobial activities against multi-drug resistant bacteria and fungi, and also degraded malachite green under ambient conditions to the tune of 93.1 %. In addition, potent blood anticoagulation and thrombolytic activities were obtained for the AgNPs. These activities have shown that the nest-mediated AgNPs can find useful biomedical and catalytic applications, particularly as antimicrobial, dye degradation, anticoagulant and thrombolytic agents. To the best of our knowledge, this is the first report of the biogenic synthesis of AgNPs using the metabolite of paper wasp, which adds to the growing utilization of novel biomaterials of arthropods in nanobiotechnology.
